# Full microscopic simulations uncover persistent quantum effects in primary photosynthesis

**DOI:** 10.1126/sciadv.ady6751

**Published:** 2025-10-01

**Authors:** Nicola Lorenzoni, Thibaut Lacroix, James Lim, Dario Tamascelli, Susana F. Huelga, Martin B. Plenio

**Affiliations:** ^1^ Institute of Theoretical Physics and IQST, Albert-Einstein Allee 11, Ulm University, 89081 Ulm, Germany.; ^2^ Dipartimento di Fisica “Aldo Pontremoli,” Università degli Studi di Milano, Via Celoria 16, 20133 Milano, Italy.

## Abstract

The presence of quantum effects in photosynthetic excitation energy transfer has been intensely debated over the past decade. Nonlinear spectroscopy cannot unambiguously distinguish coherent electronic dynamics from underdamped vibrational motion, and rigorous numerical simulations of realistic microscopic models have been intractable. Experimental studies supported by approximate numerical treatments that severely coarse-grain the vibrational environment have claimed the absence of long-lived quantum effects. Here, we report the nonperturbative, accurate microscopic model simulations of the Fenna-Matthews-Olson photosynthetic complex and demonstrate the presence of long-lived excitonic coherences at 77kelvin and room temperature, which persist on picosecond timescales, similar to those of excitation energy transfer. Furthermore, we show that full microscopic simulations of nonlinear optical spectra are essential for identifying experimental evidence of quantum effects in photosynthesis, as approximate theoretical methods can misinterpret experimental data and potentially overlook quantum phenomena.

## Teaser

Exact simulations reveal picosecond quantum coherences in the FMO photosynthetic complex at room temperature.

## INTRODUCTION

Photosynthesis is a primary biological process that converts light energy into chemical energy, playing a crucial role in sustaining life on Earth. With advancements in experimental and theoretical techniques, the structure-function relationship of photosynthetic pigment-protein complexes (PPCs) has been investigated (*1*). The electronic parameters of pigments, which absorb light and serve as excitation energy transfer (EET) channels, have been estimated using a combination of quantum chemistry methods and experimentally measured linear spectra, such as linear absorption and circular dichroism (*2*–*4*). The vibrational modes of PPCs and their interaction with electronic states are considered the primary source of environmental effects in photosynthetic EET. The phonon spectral density, which describes electronic-vibrational (vibronic) coupling strengths as a function of vibrational frequencies, is regarded as a key parameter in determining the presence of quantum effects in photosynthetic EET under ambient conditions. The phonon spectral density has been estimated using first-principles methods (*5*–*8*) and various spectroscopic techniques, such as fluorescence line narrowing (*9*).

Although the structure of microscopic models of PPCs is well understood, theoretically verifying or falsifying the presence of quantum effects, defined throughout this work as excitonic or vibronic coherences persisting on timescales comparable to those of photosynthetic EET, remains a challenging task. This difficulty arises from the comparable strengths of the electronic and vibrational parameters of PPCs, which cannot be described using perturbative theoretical methods (*10*), such as the Lindblad and Redfield equations. Despite recent advancements in nonperturbative methods (*11*–*15*), their computational costs grow substantially as the phonon spectral density becomes more structured, making numerically exact simulations of full microscopic PPC models intractable. As a result, nonperturbative simulations of PPCs have been carried out using severely coarse-grained phonon spectral densities (*16*–*19*), although these simplified noise models may underestimate quantum effects in PPCs (*10*). In this work, we show that the dissipation-assisted matrix product factorization (DAMPF) method (*13*), enhanced by incorporating thermalized spectral densities (*12*) and a systematic construction of pseudomode parameters (*15*), enables nonperturbative simulations of the full microscopic model of the Fenna-Matthews-Olson (FMO) complex without any approximations (see Supplementary Text). The DAMPF method is an alternative to other nonperturbative approaches, such as hierarchical equations of motion (HEOM) (*20*) and thermalized time-evolving density operator with orthogonal polynomials algorithm (T-TEDOPA) (*12*), as these methods yield quantitatively well-matched, numerically exact solutions of electronic dynamics and linear/nonlinear optical responses [see fig. S1G and (*10*, *12*, *13*, *15*)]. However, the enhanced DAMPF method developed in this work is currently the only practical approach for simulating the full FMO model, as HEOM and T-TEDOPA become computationally unfeasible due to the prohibitive time and memory requirements (see Materials and Methods).

Experimentally, the presence of quantum effects in PPCs has been investigated extensively, but the issue remains unresolved. To monitor EET dynamics in PPCs occurring on subpicosecond timescales, nonlinear spectroscopic techniques, such as two-dimensional electronic spectroscopy (2DES) (*21*, *22*) with femtosecond laser pulses, have been employed. These methods provide valuable insights into molecular dynamics but have a fundamental limitation in the search for quantum effects in EET. The laser pulses not only create quantum coherences between electronic excited states, associated with EET, but also induce purely vibrational coherences arising from nonequilibrium phonon dynamics in the absence of any electronic excitations, thereby not involving EET (*23*–*25*). The excited- and ground-state coherences cannot be separated using experimental techniques alone and require detailed theoretical analysis and numerical simulation. However, due to the intractability of nonperturbative simulations of PPCs using methods available at the time, the interpretation of experimental 2D spectra has relied on approximate theoretical approaches (*26*, *27*), whose failure will be shown in this work.

**Fig. 1.**
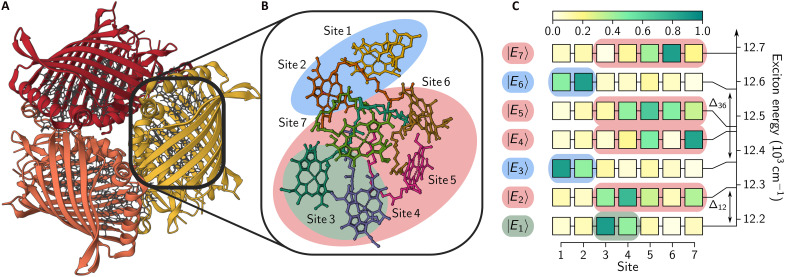
The FMO complex. (**A**) Crystal structure of the trimeric FMO complex from *Chlorobium tepidum* (*49*–*52*). (**B**) Seven BChl *a* pigments in an FMO monomer, labeled by sites 1 to 7. (**C**) Population distributions of seven exciton states $|E_{k}\rangle$ in the site basis, with $\Delta_{\mathrm{jk}}=|\;E_{j}-E_{k}\;|$ representing the energy gap between exciton states. The exciton delocalization is approximately visualized in (B). See Materials and Methods for more details.

The FMO photosynthetic complex from green sulfur bacteria, shown in Fig. 1A, has been considered in 2D experiments to investigate the presence of quantum effects (*26*–*29*). In one of the three core experimental studies suggesting the absence of long-lived excited-state coherences in the FMO complex (*30*), the 2D spectra of the FMO complex at 77 K were interpreted as follows (*27*): Excited-state coherences decay within 240fs and long-lived coherences persisting on a picosecond timescale have a vibrational origin. However, the approximate theoretical method used in (*27*) failed to explain the line shapes of experimentally measured quantum coherences, known as beating maps. In this work, we report the EET dynamics of the FMO complex at 77 and 300 K accurately computed using the full microscopic model, to show that under realistic vibrational environments, excitonic coherences in the FMO complex can persist on a picosecond timescale at both temperatures.

In another 2D study (*26*), the quantum coherences of the FMO complex measured at room temperature were attributed to vibrational origin, supported by an approximate theory using a severely coarse-grained phonon spectral density. The study of Duan *et al.* (*26*) has been considered evidence for the absence of quantum effects in the FMO complex under ambient conditions. For the coarse-grained phonon spectral density considered in (*26*), we demonstrate that the oversimplified noise model substantially underestimates the lifetime of excitonic coherences, making them hardly visible at room temperature. In addition, we report the 2D electronic spectra of model PPCs, nonperturbatively computed using the actual phonon spectral density of the FMO complex, to demonstrate that the measures in (*26*), which aim to disprove the presence of quantum effects in the FMO complex based on 2D spectra, do not provide conclusive evidence against the existence of quantum effects.

Last, FMO mutants with modified electronic parameters were studied in broadband pump probe experiments at 77 K and room temperature (*31*), revealing that the frequencies of long-lived quantum coherences on picosecond timescales remain essentially unchanged by the mutation. As approximate theoretical approaches predict that excitonic coherence frequencies should change as exciton energies are modified, the long-lived quantum coherences observed in the pump probe experiment were interpreted as vibrational in nature. In this work, we show that the frequencies of long-lived excitonic coherences are essentially invariant under variations in electronic parameters, a surprising effect induced by vibronic mixing between exciton states and the entire vibrational environment.

## RESULTS

### Microscopic model

The FMO complex serves as an EET channel between a large light-absorbing antenna complex, called chlorosome, and a reaction center where charge separation occurs. The FMO complex has a trimeric structure, as shown in Fig. 1A, and an FMO monomer consists of seven or eight pigments, depending on sample preparation, surrounded by a protein scaffold (see Fig. 1B). In molecular ensembles, the FMO complexes typically exhibit finite distributions of electronic parameters due to the static disorder induced by varying local environments. For the mean electronic parameters estimated in (*3*) (see Materials and Methods), Fig. 1C summarizes the electronic eigenstates $|\;E_{j}\;\rangle$, called excitons, delocalized over multiple pigments. The lowest energy exciton $|\;E_{1}\;\rangle$ is delocalized over sites 3 and 4, while four exciton states $|\;E_{2,4,5,7}\;\rangle$ are delocalized over multiple sites 3 to 7. The remaining exciton states $|\;E_{3}\;\rangle$ and $|\;E_{6}\;\rangle$ are delocalized over a quasi-dimeric unit of sites 1 and 2.

**Fig. 2.**
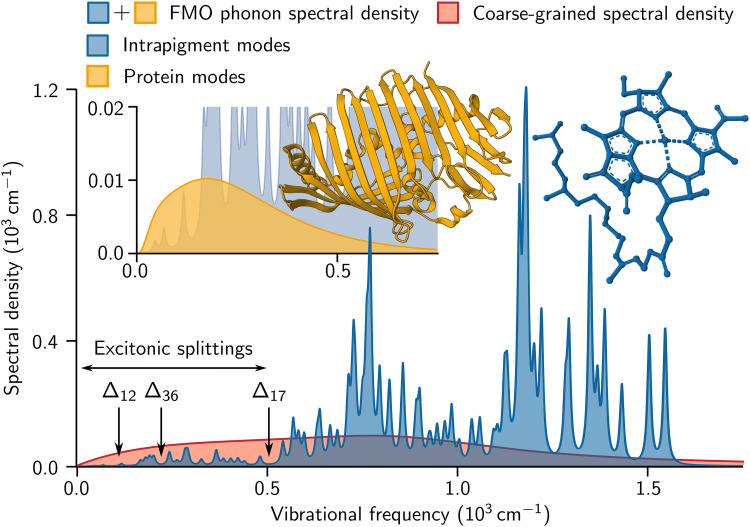
Electron-phonon coupling spectrum. The vibrational environments of the FMO complex are described by the sum of the phonon spectral density of intrapigment vibrational normal modes, shown in blue, and that of protein modes, shown in orange. This experimentally measured FMO phonon spectral density (*9*) differs substantially from the coarse-grained phonon spectral density from (*26*), shown in red. The energy differences $\Delta_{\mathrm{ij}}=|\;E_{i}-E_{j}\;|$ between exciton states $|\;E_{i}\;\rangle$ and $|\;E_{j}\;\rangle$ are indicated by black arrows, satisfying $\Delta_{\mathrm{ij}}≲500\;\;{\text{cm}}^{-1}$ (see Fig. 1C). See Materials and Methods for more details.

The vibrational environments of the FMO complex are approximately divided into two components (*9*): intrapigment and protein modes. The intrapigment vibrational normal modes exhibit a discrete frequency spectrum, resulting in several tens of narrow peaks in the phonon spectral density, as shown in blue in Fig. 2. The narrow widths indicate a low damping rate of the intrapigment vibrational modes on a picosecond timescale. In contrast, the protein modes form a quasi-continuous spectrum in the low-frequency region of the phonon spectral density, as shown in orange in Fig. 2. This experimentally estimated phonon spectral density (*9*) is quantitatively similar to those computed using first-principles methods (*6*–*8*). In addition, we consider the coarse-grained phonon spectral density from (*26*), shown in red in Fig. 2, which differs substantially from the realistic FMO phonon spectral density.

We note that an accurate description of the phonon spectral density is crucial for estimating electronic parameters from experimentally measured linear optical spectra (*10*). Here, we use the electronic parameters from (*3*), which have been widely used in FMO studies. In Supplementary Text, we provide a refined set of electronic parameters obtained by matching experimental and theoretical linear absorption and circular dichroism of the FMO complex, with the theoretical spectra computed by DAMPF based on the full FMO spectral density. For this refined parameter set, we find that the key results presented later in this work, such as the presence of long-lived excitonic coherences, remain robust (see fig. S2).

### Inter-excitonic coherences

**Fig. 3.**
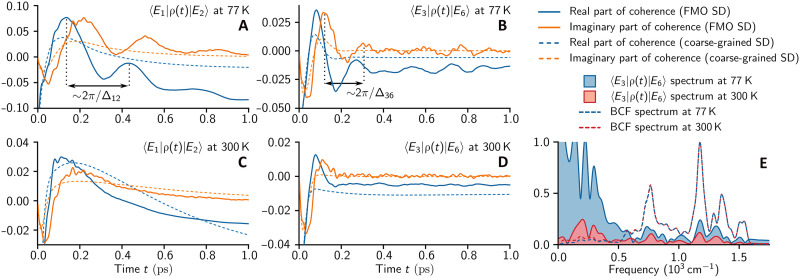
Numerically exact excitonic coherence dynamics. The real and imaginary parts of excitonic coherences $\left\langle \;E_{i}\;|\;\rho \left(t\right)\;|\;E_{j}\;\right\rangle$ are shown as functions of time t, where ρ(t) denotes the reduced electronic density matrix. The results obtained using either the microscopic FMO or the coarse-grained phonon spectral density (SD) are shown in solid and dashed lines, respectively (see Fig. 2). Simulations were performed for the following cases: (**A**) (i,j)=(1,2) at 77K, (**B**) (i,j)=(3,6) at 77K, (**C**) (i,j)=(1,2) at 300K, and (**D**) (i,j)=(3,6) at 300 K. (**E**) The frequency spectra of the long-lived excitonic coherence dynamics shown in (B) and (D) over a time window 300fs≤t≤1ps are presented, along with the frequency spectra of the bath correlation functions (BCFs) at 77 and 300 K.

Using numerically exact nonperturbative methods, we simulated excitonic coherence dynamics in the FMO complex for an initial state where all seven exciton states are superposed with similar amplitudes, a state that is a typical result of laser excitation (see Materials and Methods). In Fig. 3, we present excitonic coherence dynamics under the influence of either the FMO or the coarse-grained phonon spectral density from (*26*) (see Fig. 2).

Figure 3A shows the coherence dynamics between the two lowest-energy exciton states $|\;E_{1}\;\rangle$ and $|\;E_{2}\;\rangle$ at 77 K, which has been the primary focus of 2D experiments. For the FMO phonon spectral density, the oscillatory coherence dynamics persist on a picosecond timescale, demonstrating that the lifetime of excitonic coherences at 77 K was severely underestimated in (*27*). At 77 K, long-lived coherences were also found for other exciton pairs. As an example, Fig. 3B shows the coherence dynamics between exciton states $|\;E_{3}\;\rangle$ and $|\;E_{6}\;\rangle$. Note that the numerically exact excitonic coherence dynamics exhibit multiple frequencies. While the frequency of the large-amplitude oscillations is close to the energy gap $\Delta_{\mathrm{ij}}=|\;E_{i}-E_{j}\;|$ between the exciton states, high-frequency oscillatory components with relatively small amplitudes are present in both Fig. 3 (A and B). These high-frequency features disappear when the coarse-grained spectral density is considered.

At room temperature, while the $\Delta_{\mathrm{ij}}$-frequency oscillations decay more quickly within 300 fs, the long-lived high-frequency oscillations observed at 77 K are preserved, as shown in Fig. 3 (C and D). Note that the coarse-grained spectral density completely suppresses all oscillatory features in the excitonic coherence dynamics at 300 K, substantially underestimating quantum effects in the FMO complex under realistic vibrational environments.

To analyze the nature of the long-lived quantum coherences, Fig. 3E presents the frequency spectrum of the excitonic coherence dynamics between $|\;E_{3}\;\rangle$ and $|\;E_{6}\;\rangle$ over a time window from 300fs to 1ps. At both 77 and 300K, narrow peaks appear across the entire vibrational frequency range of the intrapigment modes. A broad peak feature below $500\;{\text{cm}}^{-1}$ is observed only at 77 K, where $\Delta_{36}$-frequency oscillations persist beyond 300 fs, unlike at room temperature. We note that the narrow-peak structure is similar to that of the frequency spectrum of the bath correlation function (BCF) (*32*), describing the influence of the FMO phonon spectral density on excitonic dynamics over the simulated picosecond timescale (see Materials and Methods) (*15*). This suggests that phonon-mediated transitions between the exciton states occur not only via resonant vibrational modes with frequencies close to the electronic energy gap $\Delta_{36}$ but also through interactions with the entire vibrational environment (*10*). Thus, vibronic interactions in the FMO complex go beyond the weak coupling regime, and long-lived excitonic coherences are supported by the entire highly-structured phonon spectral density, instead of a few resonant vibrational modes, revealing the limitations of conventional theoretical approaches (*24*, *33*, *34*).

To clarify the physical origin of the long-lived excitonic coherences in our simulations, we describe a mechanism based on two well-established properties of photosynthetic systems. Even in the perturbative regime, the ratio between the energy gap and the coupling between quantum states serves as a quantifier of the degree of interaction. In the case of photosynthetic PPCs, the energy gap between a higher-energy exciton state with all the intrapigment modes in their vibrational ground states and a lower-energy exciton state with one intrapigment mode singly excited is given by $|\;\Delta -\omega_{k}\;|$, where Δ>0 denotes exciton energy difference, and $\omega_{k}$ represents the vibrational frequency of the excited mode. The coupling between them is proportional to the vibronic coupling creating a single vibrational excitation, given by $\omega_{k}\sqrt{s_{k}}$, where $s_{k}$ is the so-called Huang-Rhys factor. The first property of photosynthetic systems composed of chlorophylls or bacteriochlorophylls (e.g., the FMO complex) is that the Huang-Rhys factors are nearly independent of the vibrational frequencies $\omega_{k}$ of intrapigment modes and are typically on the order of 0.01 (see table S3). This implies that for small excitonic splittings Δ, the detuning $|\;\Delta -\omega_{k}\;|$ increases linearly with $\omega_{k}$, so does the coupling $\omega_{k}\sqrt{s_{k}}\approx 0.1\omega_{k}$, making the ratio between detuning and coupling nearly independent of $\omega_{k}$. Therefore, this nearly frequency-independent nature of the Huang-Rhys factors of photosynthetic complexes can result in nontrivial interaction between excitons and the entire set of intrapigment vibrational modes.

The second property of photosynthetic systems, which induces nontrivial electronic-vibrational interactions, is that the vibronic couplings are beyond the weak coupling regime, as their magnitude is comparable to that of the electronic couplings between pigments. In this beyond-weak-coupling regime, the concept of a resonance condition, where excitons mainly interact with near-resonant vibrational modes satisfying $\Delta \approx \omega_{k}$, is not valid. As a result, the eigenstates of photosynthetic complexes can exhibit nontrivial superpositions involving multiple exciton states and vibrational modes where resonance conditions are not satisfied (*10*). This multimode vibronic mixing has been demonstrated in detail for dimeric PPCs, such as the water-soluble chlorophyll-binding protein from cauliflower and the special pair unit of bacterial reaction centers from purple bacteria, using nonperturbative HEOM and T-TEDOPA methods (*10*). The multifrequency nature of the excitonic coherences in the FMO complex, as presented in this work, demonstrates that the multimode vibronic mixing can also occur in multichromophoric photosynthetic complexes. This mechanism therefore demonstrates that excitonic states can couple nonresonantly to a broad range of vibrational modes, potentially leading to persistent quantum coherences not captured in theoretical models that use a simplified form of the spectral density.

**Fig. 4.**
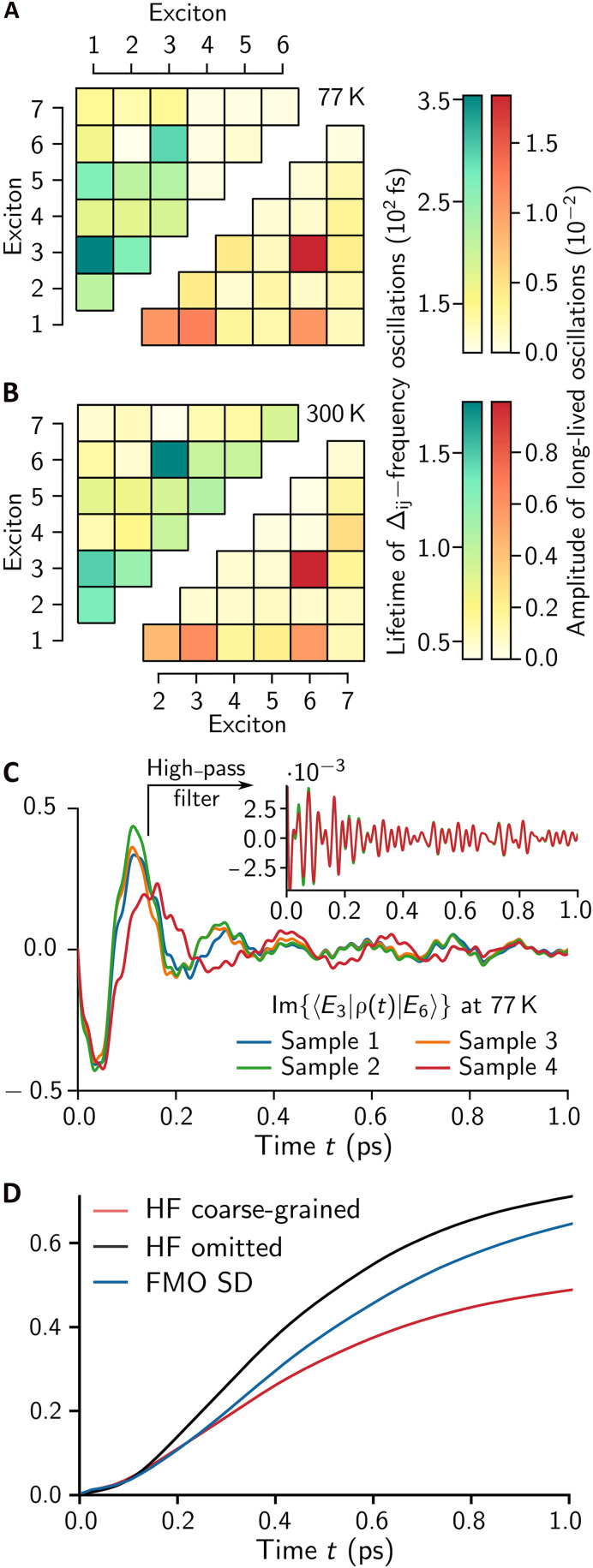
Long-lived excitonic coherences and their robustness against static disorder. The lifetimes of the large-amplitude oscillations with frequencies $\Delta_{\mathrm{ij}}$ and the amplitudes of long-lived multifrequency oscillations in the excitonic coherence dynamics $\left\langle \;E_{i}\;|\;\rho \left(t\right)\;|\;E_{j}\;\right\rangle$ are shown at (**A**) 77 K and (**B**) 300 K. (**C**) The excitonic coherence dynamics $\left\langle \;E_{3}\;|\;\rho \left(t\right)\;|\;E_{6}\;\right\rangle$ between $|\;E_{3}\;\rangle$ and $|\;E_{6}\;\rangle$ at 77 K, computed with four randomly generated sets of electronic parameters based on a Gaussian static disorder model, are shown. The coherence dynamics exhibit essentially identical high-frequency oscillations, as shown in the inset, obtained via a high-pass filter. (**D**) For the initial state $|E_{7}\rangle$, the population dynamics of $|\;E_{1}\;\rangle$ at 77 K are present under three different phonon spectral densities (SDs): The microscopic FMO SD and two model SDs in which high-frequency (HF) intrapigment modes beyond $700\;{\text{cm}}^{-1}$ are either omitted or coarse-grained. See Materials and Methods for more details.

For all possible exciton pairs (j,k), Figs. 4A and B summarize the lifetimes of the large amplitude oscillations of excitonic coherences with frequencies close to $\Delta_{\mathrm{jk}}$, along with the amplitudes of high-frequency long-lived oscillations. We found that the coherence between exciton states exhibits the long-lived multifrequency characteristics when at least one of the states is either the lowest-energy exciton $|\;E_{1}\;\rangle$ or the meta-stable state $|E_{3}\rangle$ whose population transfer to other exciton states remains small within 1 ps (see fig. S3A). This observation was further confirmed by modifying electronic parameters, varying the number of meta-stable states (see Supplementary Text and fig. S3). Notably, we found that while the frequencies of the large-amplitude oscillations depend on the randomness of electronic parameters induced by static disorder, the high-frequency oscillations remain nearly unchanged, as shown in Fig. 4C. These results indicate that long-lived excitonic coherences supported by multimode vibrational environments of PPCs do not require fine-tuning of electronic energy-level structures, making the observed quantum effects relevant in biological conditions.

We note that intrapigment modes with frequencies beyond the energy range of excitonic splittings $\left(\Delta_{\mathrm{ij}}<700\;{\text{cm}}^{-1}\right)$ can substantially influence EET, although high-frequency components of excitonic coherences exhibit small amplitudes. This is due to quantum effects in photosynthetic EET arising from superpositions between electronic-vibrational states of photosynthetic excitons and intrapigment vibrational modes, which are only partially revealed by monitoring excitonic coherence dynamics. Figure 4D presents the population dynamics of the lowest-energy exciton $|\;E_{1}\;\rangle$ when the highest-energy exciton $|E_{7}\rangle$ is initially populated. In addition to the full FMO phonon spectral density, we examine two model spectral densities in which protein and intrapigment modes up to $700\;{\text{cm}}^{-1}$ are treated accurately, while all other high-frequency intrapigment modes are either omitted or coarse-grained with an increased vibrational damping rate ${\left(50\;\text{fs}\right)}^{-1}$ (see fig. S4). The population dynamics show notable differences depending on how these high-frequency modes are handled, providing further evidence that the multimode vibronic mixing occurs in the FMO complex.

### 2D electronic spectroscopy

So far, we have theoretically demonstrated that excitonic coherences in the FMO complex are long-lived in realistic vibrational environments. To clarify that our results do not contradict the experimental evidence used to claim the absence of quantum effects in the FMO complex, we revisit the measures discussed in (*26*).

In 2D electronic spectroscopy (*21*, *22*), the third-order molecular polarization induced by a sequence of laser pulses is measured as a function of time delay $t_{2}$ between pump and probe pulses. By using a pair of pump pulses with controlled time delay, 2D electronic spectra are measured as a function of excitation and detection frequencies $\left(\omega_{1},\omega_{3}\right)$ for each waiting time $t_{2}$. In particular, for the rephasing 2D electronic spectra at $t_{2}=0$, the width of diagonal peaks in the antidiagonal direction allows an estimation of the lifetime of optical coherence (i.e., between the electronic ground and excited states) at the single-molecule level, thereby separating the homogeneous broadening from the inhomogeneous broadening caused by static disorder. In (*26*), supplied by an approximate theoretical approach using the coarse-grained spectral density shown in Fig. 2, the experimentally measured lifetime of optical coherences around 60 fs was interpreted as the lifetime of excitonic coherences and presented as one piece of evidence supporting the absence of quantum effects in the FMO complex at room temperature. However, the lifetime of optical coherences does not directly determine that of excitonic coherences, as the relationship between them depends strongly on the nature of the electron-phonon interaction.

**Fig. 5.**
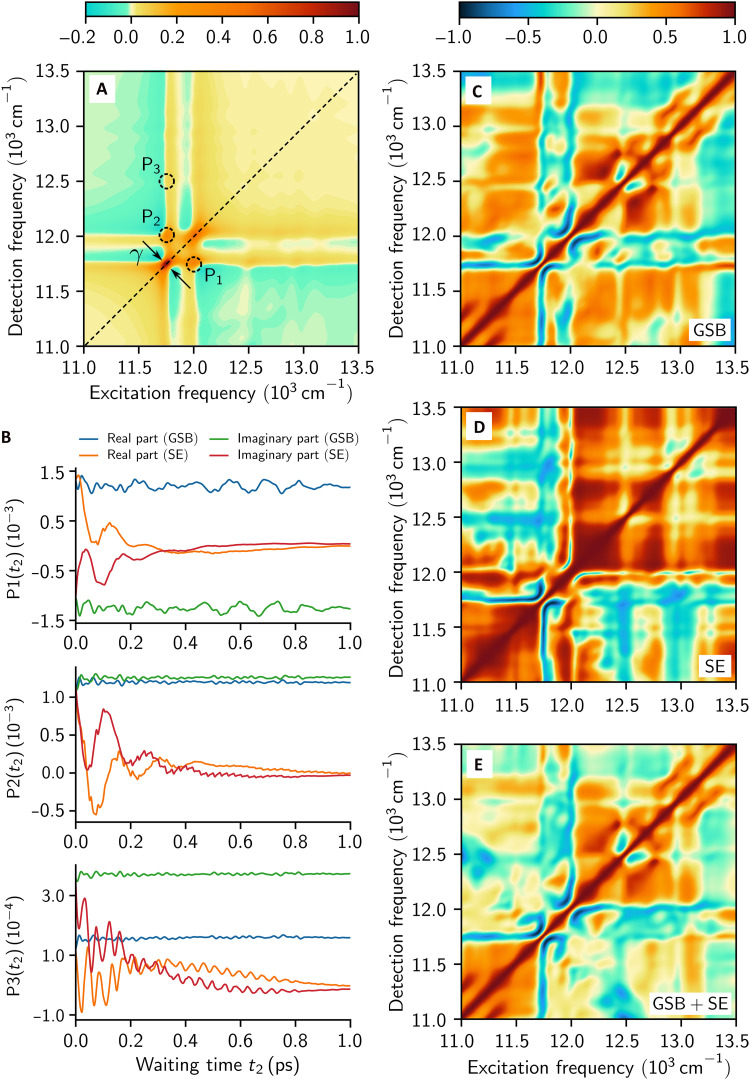
Numerically exact 2D electronic spectra. (**A**) The rephasing spectra of a heterodimer at $t_{2}=0$ are shown with γ denoting the antidiagonal width of a diagonal peak. (**B**) For the three peak positions marked in (A), the peak dynamics as functions of the waiting time $t_{2}$ are displayed. The correlation maps of (**C**) GSB, (**D**) SE, and (**E**) the total 2D signals (GSB + SE) are shown, computed based on the real parts of the long-lived oscillatory signals over a finite time window of $300\;\text{fs}\leq t_{2}\leq 1\;\text{ps}$. In simulations, the microscopic FMO phonon spectral density at 77 K was considered (see Fig. 2). See Materials and Methods for more details.

To clarify this issue, we simulated the rephasing 2D spectra of a dimeric PPC model at 77 K, computed nonperturbatively using the FMO phonon spectral density (see Materials and Methods). The total 2D spectra are expressed as the sum of the stimulated emission (SE) and ground-state bleaching (GSB) signals, which provide information about the molecular dynamics within the electronic excited-state and ground-state manifolds, respectively, during the waiting time $t_{2}$ (*22*, *35*, *36*). While the SE and GSB signals can be computed separately, only the total signal can be measured in experiments. Therefore, by comparing the lifetime of oscillatory SE signals, originating from excited-state coherences, with the antidiagonal width of a diagonal peak in rephasing 2D spectra at $t_{2}=0$, one can verify whether the experimental measure considered in (*26*) is reliable. Figure 5A displays the numerically exact rephasing spectra at $t_{2}=0$, where the antidiagonal width is ~200fs. As shown in Fig. 5B, the oscillatory features in the SE signals last up to 1ps, demonstrating that the lifetime of excited-state coherences appearing in 2D spectra is not limited by the antidiagonal width.

As another evidence, Duan *et al.* (*26*) examined the correlation between oscillatory 2D signals for each pair of spectral positions $\left(\omega_{1},\omega_{3}\right)$ and $\left(\omega_{3},\omega_{1}\right)$, which are symmetric with respect to the diagonal line $\omega_{1}=\omega_{3}$. The relative phase between these oscillations can be quantified using the Pearson correlation coefficient, where positive and negative values indicate in-phase and out-of-phase relationships, respectively. On the basis of approximate theoretical results (*23*), it was hypothesized that negative correlations serve as signatures of ground-state coherences, since excited-state coherences were expected to produce only positive correlations. However, this hypothesis does not hold for realistic PPC models. Figure 5 (C and D) present the correlation maps of the GSB and SE signals, respectively. Both maps display regions of positive and negative correlations depending on spectral positions, indicating that negative correlations do not necessarily imply the oscillatory GSB signals originating from ground-state coherences. As shown in Fig. 5E, the correlation map of the total 2D spectra exhibits negative correlations at cross peaks and positive correlations at diagonal peaks. This pattern closely resembles the experimentally measured correlation map of the FMO complex reported in (*26*). Since long-lived excited-state coherences can be present at the negatively correlated cross peaks [see Fig. 5 (B and E) and fig. S5], the negative correlations cannot be taken as evidence for the absence of excited-state coherences.

## DISCUSSION

Our results demonstrate that quantum effects in the FMO complex persist up to 1ps and beyond, which is the timescale of photosynthetic EET. These conclusions are based on electronic dynamics rigorously computed using a microscopic model constructed from first-principles methods (*6*–*8*) and validated with experimental spectroscopic data, such as linear absorption, linear and circular dichroism (*2*, *3*), and fluorescence line narrowing (*9*). More broadly, our findings suggest that similar quantum effects may exist in other PPCs, as highly structured phonon spectral densities are a ubiquitous feature arising from the vibrational normal modes of pigments (*37*–*40*).

Our 2D simulations indicate that verifying or falsifying quantum effects in PPCs cannot be reliably achieved using nonlinear spectroscopic data analyzed with approximate theoretical methods. While various nonlinear spectroscopic techniques have been designed to isolate excited-state coherences, such as through controlled laser pulse polarizations (*27*) or colors (*41*), their performance in eliminating ground-state coherences remains insufficient for investigating quantum effects in PPCs (*36*). Therefore, a quantitative, rather than qualitative, analysis of nonlinear spectroscopic data using microscopic PPC models is essential for isolating excited-state coherences from experimental results, which are often contaminated by ground-state coherences, an approach that has not yet been explored in current state-of-the-art research.

Alternatively, one could examine spectral regions of 2D spectra where the ground-state coherences are minimal. For instance, nonperturbative simulations of the dimeric system coupled to the full FMO spectral density, presented in this work, show that the oscillatory components in the GSB signals are confined to the frequency range of intrapigment modes (i.e., $≲1600\;{\text{cm}}^{-1}$). This is due to the small Huang-Rhys factors of the intrapigment modes of bacteriochlorophylls in the FMO complex, which induce notable single-phonon transitions (e.g., 0 to 1) but only negligible two-phonon transitions (e.g., 0 to 2). We found that the oscillatory components in the SE signals extend well beyond the frequency range of the intrapigment modes (i.e., $\gg \;1600\;{\text{cm}}^{-1}$; see fig. S6). While it remains uncertain whether this feature persists in multichromophoric systems beyond dimers and under static disorder, detecting such high-frequency 2D oscillations experimentally could motivate further theoretical analysis. This, in turn, may help determine whether long-lived excitonic or vibronic coherences are present in PPCs.

While unambiguous identification of long-lived coherent electronic dynamics via nonlinear spectroscopy remains challenging, quantum control schemes using sequences of infrared and visible pulses (*42*–*44*) may offer a promising route to test the proposed multimode vibronic mechanism. Perturbing high-frequency vibrational modes, whose frequencies exceed exciton energy splittings (~700 cm^−1^ in the case of the FMO complex), using an infrared pulse before measuring linear and nonlinear optical responses via visible pulses may reveal whether these high-frequency modes can influence electronic dynamics. If only vibrational modes near-resonant with exciton energy differences contribute, and others can be considered spectator modes, such perturbation is expected to have marginal impact. However, if the entire vibrational environment plays a role, as suggested by our simulations, then the infrared preexcitation may alter the energy gaps between peaks in linear and nonlinear optical spectra or modify the timescales of nonoscillatory (exponential) components in nonlinear signals, such as pump-probe and 2D electronic spectra, that reflect transfer rates between exciton states. Thus, such an infrared-visible control scheme may provide a means to experimentally verify or falsify the multimode vibronic mechanism responsible for the long-lived excitonic coherences observed in our simulations. In addition, the development of new experimental techniques may be required, as most nonlinear spectroscopic techniques measure the optical responses of molecular ensembles and may underestimate quantum effects in photosynthetic EET occurring at the single-molecule level.

Ultimately, theoretical studies of full microscopic PPC models will provide a new framework for interpreting experimental data on photosynthetic PPCs, where electronic dynamics occur in nonperturbative regimes. This may reveal how photosynthetic EET occurs in real biological environments and how quantum effects are sustained and utilized under ambient conditions.

## MATERIALS AND METHODS

### Microscopic model of the FMO photosynthetic complex

The Hamiltonian of the FMO complex is modeled by $H=H_{e}+H_{v}+H_{e-v}$, where $H_{e}$ is the electronic Hamiltonian, $H_{v}$ is the vibrational Hamiltonian, and $H_{e-v}$ describes the interaction Hamiltonian between electronic states and vibrational modes. Each pigment is modeled as a two-level system, as the energy gap between its first and second excited states is sufficiently large to allow selective excitation of the first excited state using a laser pulse. We consider the electronic ground and singly excited state manifolds, as these states govern photosynthetic EET, and the linear and nonlinear optical spectra.

The electronic Hamiltonian is given by

$$\begin{array}{c} H_{e}=\sum_{n=1}^{7}\epsilon_{n}\;|\;\epsilon_{n}\rangle \langle \epsilon_{n}\;|+\sum_{n\neq m}^{7}V_{\mathrm{nm}}\;|\;\epsilon_{n}\rangle \langle \epsilon_{m}\;| \end{array}$$ (1)

where $|\;\epsilon_{n}\;\rangle$ represents the local electronic excitation at site n with site energy $\epsilon_{n}$, while $V_{\mathrm{nm}}$ denotes the electronic coupling between sites n and m. Here, we consider the electronic parameters of the FMO complex estimated in (*3*), summarized in table S1.

The vibrational modes are modeled as independent quantum harmonic oscillators, with the vibrational Hamiltonian

$$H_{v}=\sum_{n=1}^{7}\sum_{\xi }\omega_{\xi }b_{n,\xi }^{†}b_{n,\xi }$$ (2)

where $b_{n,\xi }^{†}$ and $b_{n,\xi }$ are the creation and annihilation operators of the vibrational mode with frequency $\omega_{\xi }$, locally coupled to site n. The interaction Hamiltonian is expressed as

$$\begin{array}{c} H_{e-v}=\sum_{n=1}^{7}|\;\epsilon_{n}\rangle \langle \epsilon_{n}\;|\sum_{\xi }\omega_{\xi }\sqrt{s_{\xi }}\left(b_{n,\xi }+b_{n,\xi }^{†}\right) \end{array}$$ (3)

where the vibronic coupling strength is quantified by the Huang-Rhys factor $s_{\xi }$.

In this work, we present excitonic population and coherence dynamics, obtained by nonperturbatively simulating $\rho \left(t\right)={\text{Tr}}_{v}\left[e^{-\mathrm{iHt}}\rho \left(0\right)\rho_{v}\left(T\right)e^{\mathrm{iHt}}\right]$, where ρ(t) is the reduced electronic density matrix at time t, ${\text{Tr}}_{v}$ denotes the partial trace over vibrational degrees of freedom, ρ(0) is the initial electronic state, and $\rho_{v}\left(T\right)=\text{exp}\left(\;-H_{v}/k_{B}T\right)/\text{Tr}\left[\text{exp}\left(\;-H_{v}/k_{B}T\right)\right]$ is the thermal state of the vibrational environment at temperature T. In the excitonic coherence simulations, shown in Figs. 3 and 4C, the initial electronic state is modeled as ρ(0)=∣ψ⟩⟨ψ∣ with $|\;\psi \rangle \propto \sum_{n=1}^{7}\left(\mu_{n}\cdot e_{\text{Laser}}\right)|\epsilon_{n}\rangle$, where $\mu_{n}$ denotes the transition dipole moment of site n in the FMO complex, summarized in table S2, and $e_{\text{Laser}}=\left(0.25,0.85,0.0\right)$ represents the laser pulse polarization, chosen to populate all seven exciton states $|\;E_{k}\;\rangle$ with comparable magnitudes. In the exciton population simulations shown in Fig. 4D, the initial state is assumed to be the highest-energy exciton state, $|\;\psi \;\rangle =|\;E_{7}\;\rangle$.

The influence of the vibrational environment on the reduced electronic density matrix ρ(t) is fully characterized by the phonon spectral density defined as $J\left(\omega \right)=\sum_{\xi }\omega_{\xi }^{2}s_{\xi }\delta \left(\omega -\omega_{\xi }\right)$. The experimentally estimated phonon spectral density of the FMO complex (*9*) is given by $J\left(\omega \right)=J_{\text{AR}}\left(\omega \right)+J_{L}\left(\omega \right)$, where $J_{\text{AR}}\left(\omega \right)$ represents the Adolphs-Renger (AR) spectral density describing protein modes

$$J_{\text{AR}}\left(\omega \right)=\frac{S_{0}}{S_{1}+S_{2}}\sum_{i=1}^{2}\frac{S_{i}}{7!2\Omega_{i}^{4}}\omega^{5}e^{-{\left(\omega /\Omega_{i}\right)}^{1/2}}$$ (4)

with $S_{0}=0.29$, $S_{1}=0.8$, $S_{2}=0.5$, $\Omega_{1}=0.069\;\text{meV}$, and $\Omega_{2}=0.24\;\text{meV}$, while $J_{L}\left(\omega \right)$ denotes the sum of 62 Lorentzian spectral densities corresponding to intrapigment modes

$$\begin{array}{c} J_{L}\left(\omega \right)=\sum_{k=1}^{62}\frac{4\omega_{k}s_{k}\gamma_{k}\left(\omega_{k}^{2}+\gamma_{k}^{2}\right)\omega }{\pi \left[{\left(\omega +\omega_{k}\right)}^{2}+\gamma_{k}^{2}\right]\left[{\left(\omega -\omega_{k}\right)}^{2}+\gamma_{k}^{2}\right]} \end{array}$$ (5)

Each Lorentzian spectral density is characterized by the vibrational frequency $\omega_{k}$ and Huang-Rhys factor $s_{k}$ of the kth intrapigment mode, as summarized in table S3. The vibrational damping rates of the intrapigment modes are taken to be $\gamma_{k}={\left(1\;\text{ps}\right)}^{-1}\approx 5\;{\text{cm}}^{-1}$. The reorganization energy of the intrapigment modes is given by $\int_{0}^{\infty }d\omega J_{L}\left(\omega \right)/\omega =\sum_{k=1}^{62}\omega_{k}s_{k}$, which is independent of the vibrational damping rates $\gamma_{k}$. The total reorganization energy of the FMO phonon spectral density J(ω) is $\int_{0}^{\infty }d\omega J\left(\omega \right)/\omega \approx 232\;{\text{cm}}^{-1}$.

### Model phonon spectral densities with approximated high-frequency intrapigment modes

In Fig. 4D, we consider two model spectral densities where the first 26 intrapigment modes with $\omega_{k}<700\;{\text{cm}}^{-1}$ are considered accurately using the parameters in table S3 with $\gamma_{k}={\left(1\;\text{ps}\right)}^{-1}$, while the other high-frequency intrapigment modes are either omitted ($s_{k}=0$ for k>26) or coarse-grained ($\gamma_{k}={\left(50\;\text{fs}\right)}^{-1}$ for k>26), as shown in black and red, respectively, in fig. S4. In both cases, the AR spectral density $J_{\text{AR}}\left(\omega \right)$ is considered. The total reorganization energy of the coarse-grained case is identical to that of the FMO phonon spectral density.

### Coarse-grained phonon spectral density

In Figs. 2 and 3, we consider the coarse-grained phonon spectral density from (*26*)

$$J_{\text{CG}}\left(\omega \right)=\frac{2}{\pi }\gamma \omega e^{-\frac{\omega }{\omega_{c}}}+\frac{4}{\pi^{2}}S\Omega^{3}\frac{\omega \Gamma }{{\left(\Omega^{2}-\omega^{2}\right)}^{2}+\omega^{2}\Gamma^{2}}$$ (6)

where γ=0.7, $\omega_{c}=350\;{\text{cm}}^{-1}$, S=0.12, $\Omega =900\;{\text{cm}}^{-1}$, and $\Gamma =700\;{\text{cm}}^{-1}$, resulting in a reorganization energy of ~$224\;{\text{cm}}^{-1}$. We note that these parameters were confirmed by the first author of (*26*) through private communication.

### Refined electronic parameters of the FMO complex

The electronic parameters of the FMO complex have been estimated using first-principles methods and fine-tuned based on a comparison of theoretically computed linear optical spectra with experimental data, such as linear absorption and circular dichroism (*3*). However, the linear optical spectra have been computed using approximate methods, thereby a refinement of electronic parameters based on nonperturbative simulations is needed (*10*).

Figure S2A shows the numerically exact absorption and circular dichroism spectra at T=77 K, computed based on of a refined set of electronic parameters summarized in table S4. Here, the electronic parameters were optimized to quantitatively reproduce the experimental data.

For the refined set of electronic parameters (i.e., table S4), fig. S2B summarizes the properties of exciton states (i.e., the electronic eigenstates of $H_{e}$), which are qualitatively similar to those shown in Fig. 1C, obtained using the electronic parameters estimated in (*3*) (i.e., table S1).

In fig. S2D, the excitonic coherence dynamics between $|\;E_{1}\;\rangle$ and $|\;E_{2}\;\rangle$ ($|\;E_{3}\;\rangle$ and $|\;E_{6}\;\rangle$) at 77 K, nonperturbatively computed based on the refined set of electronic parameters, are shown in solid lines. For comparison with Fig. 3, the excitonic coherence dynamics computed using the electronic parameters estimated in (*3*) are shown as dashed lines. For both sets of electronic parameters, the excitonic coherences exhibit large-amplitude oscillations with a frequency close to the energy-gap $\Delta_{\mathrm{ij}}=|\;E_{i}-E_{j}\;|$ between exciton states, along with high-frequency long-lived oscillations of relatively small amplitudes. Notably, the high-frequency long-lived components of the excitonic coherence between $|\;E_{3}\;\rangle$ and $|\;E_{6}\;\rangle$ are nearly identical for both parameter sets. This is illustrated in fig. S2E, where a high-frequency filter was applied to remove the large-amplitude oscillations associated with the lower-frequency $\Delta_{36}$. In addition to Fig. 4C, fig. S2E further demonstrates that the long-lived excitonic coherences of the FMO complex are robust against variations in electronic parameters.

### Nonperturbative simulation method

Nonperturbatively simulating the dynamics of open quantum systems at finite temperatures is a formidable challenge in the presence of highly structured spectral densities, as those typical of PPCs. In this work, we tackle this scenario by using the DAMPF method (*13*), a nonperturbative, numerically exact approach that has been successfully applied to the simulation of vibronic systems (*45*). We further enhance it by incorporating the systematic coarse-graining of environments introduced in (*15*) and the concept of thermalized spectral density from (*12*).

In particular, since the electronic degrees of freedom linearly couple to a collection of quantum harmonic oscillators representing the vibrational environment of PPCs, and the thermal state $\begin{array}{c} \rho_{v}\left(T\right)=\text{exp}\left(\;-H_{v}/k_{B}T\right)/\text{Tr}\left[\text{exp}\left(\;-H_{v}/k_{B}T\right)\right] \end{array}$ is taken as the initial state of the environment, it follows from (*46*) that the original environmental modes can be replaced by a finite number of damped quantum harmonic oscillators (pseudomodes), as both yield an identical reduced electronic density matrix ρ(t), provided that their two-time BCFs are well matched (*13*). Since energy transfer in the FMO complex occurs on a characteristic timescale of 1 ps, we perform numerically exact simulations of excitonic population and coherence dynamics of the FMO complex up to 1 ps. This allows us to apply the systematic environmental coarse-graining technique of (*15*), enabling the use of a simplified, effective environment that accurately reproduces the BCF of the FMO phonon spectral density, defined as $\text{BCF}\left(t\right)=\int_{0}^{\infty }d\omega J\left(\omega \right)\left[\text{coth}\left(\omega /2k_{B}T\right)\text{cos}\left(\omega \;t\right)-i\;\text{sin}\left(\omega \;t\right)\right]$, over the finite time window 0≤t≤1 ps. In fig. S1A, we show the frequency spectrum of the BCF at T=77 K of the effective environment modeled using 31 pseudomodes for DAMPF simulations. Figure S1B demonstrates that the BCFs of the FMO phonon spectral density and the effective pseudomode environment are well matched up to 1 ps. This ensures that the reduced electronic density matrix ρ(t) can be computed accurately within the finite time window 0≤t≤1 ps using DAMPF.

For nonperturbative simulations of 2D rephasing spectra of a dimeric PPC model at T=77 K, we considered an extended time window 0≤t≤1.5 ps, leading to an effective environment modeled using 35 pseudomodes, as shown in fig. S1A, which accurately reproduces the BCF of the FMO phonon spectral density up to 1.5 ps, as shown in fig. S1B. In 2D simulations, light-matter interaction was treated perturbatively to compute the GSB and SE signals separately. This assumption is valid since the laser intensity is reduced in 2D experiments until the rephasing spectra show convergence, ensuring that experimental data are dominated by third-order optical responses with negligible higher-order contributions. We considered a heterodimer with a site energy difference of $\epsilon_{1}-\epsilon_{2}=200\;{\text{cm}}^{-1}$ and an intersite electronic coupling of $V_{12}=100\;{\text{cm}}^{-1}$. The transition dipole moments of the two pigments were assumed to be orthogonal, similar to sites 1 and 2 of the FMO complex, and an orientational average with respect to the fixed laser polarization was taken into account in simulations. As in the case of the FMO complex, we assumed that each site of the dimer is coupled to a local vibrational environment modeled by the FMO phonon spectral density.

At room temperature T=300 K, DAMPF simulations become substantially more challenging due to the increased local dimensions of the pseudomodes caused by higher thermal populations. To achieve the same level of accuracy as in the T=77 K simulations, we improved the DAMPF method by incorporating the concept of thermalized spectral density introduced in (*12*). This approach effectively describes a finite-temperature harmonic environment using a zero-temperature harmonic environment while accurately capturing the influence of finite temperature on the reduced open system dynamics, at the cost of enhanced couplings and the introduction of negative-frequency harmonic modes. When this idea is directly applied to DAMPF, one can consider zero-temperature pseudomodes with either positive or negative frequencies. However, at lower temperatures, correlations between pseudomodes tend to increase, which can lead to higher bond dimensions and increased computational costs. Thus, there exists an optimal temperature range where the thermal populations of the pseudomodes are lower than at room temperature, while the correlations between pseudomodes are lower than at zero temperature. In this work, we considered a partial thermal spectral density by independently optimizing the temperatures of the pseudomodes, along with their frequencies, decay rates, and the Huang-Rhys factors when fitting the BCF of the FMO phonon spectral density at T=300 K. As in the case of the full thermalized spectral density (*12*), the partial thermal spectral density increases the couplings between electronic states and pseudomodes and requires the use of negative frequencies for some pseudomodes, but both the local and bond dimensions are substantially reduced, boosting numerical efficiency. In fig. S1C, we show the frequency spectrum of the BCF at T=300 K of the effective environment modeled using 33 pseudomodes for DAMPF simulations, including two modes with negative frequencies. Figure S1D demonstrates that the BCFs are well matched up to 1 ps, ensuring that the room temperature EET dynamics of the FMO complex can be simulated with high accuracy using DAMPF.

To test the accuracy of the pseudomode parameters prepared for T=77 K and T=300 K DAMPF simulations of the FMO complex, we compared analytical solutions of the optical coherence dynamics of a monomer, where a single site couples to the FMO phonon spectral density, with numerical results obtained by DAMPF. As shown in fig. S1 (E and F), the numerically exact monomer dynamics obtained by DAMPF for T=77 K and T=300 K are well matched with the analytical solutions.

To further demonstrate the accuracy of our DAMPF method, fig. S1G shows the excitonic coherence dynamics of a dimeric system with each site subject to the full FMO phonon spectral density, simulated using DAMPF and the T-TEDOPA (*12*, *47*, *48*). In the simulations, we considered a site energy difference of $\epsilon_{1}-\epsilon_{2}=100\;{\text{cm}}^{-1}$ and an intersite electronic coupling of $V_{12}=50\;{\text{cm}}^{-1}$, with the higher-energy site initially excited. The results obtained from the two nonperturbative methods are quantitatively well-matched, confirming the reliability of the DAMPF results presented in this work. Due to the high computational cost of T-TEDOPA, the simulation times were limited to 500 fs at 77 K and 250 fs at 300 K. On the same hardware (48 cores on Intel Xeon 6252 Gold CPUs), the T-TEDOPA required 18 hours for the 77 K simulation and 50 hours for the 300 K simulation, whereas DAMPF completed both in just 5 min, highlighting the substantial computational advantage of DAMPF.

The HEOM method (*20*) is widely considered the gold standard for nonperturbative simulations of photosynthetic complexes. However, its computational cost is even greater than that of T-TEDOPA, making simulations of the dimeric system with each site subject to the full FMO spectral density challenging. For a PPC model consisting of N pigments coupled to local vibrational environments modeled by M Lorentzian spectral densities, the total number of auxiliary operators of the HEOM method up to the Lth hierarchical layer is given by (2NM+L)!/(2NM)!/L! when the Matsubara terms are not considered. Assuming a hierarchical depth of L=10, required to account for underdamped intrapigment modes of PPCs (*10*), a comparison of the memory costs of HEOM and DAMPF simulations for N=7 pigments (e.g., the FMO complex) is presented in fig. S1H. Notably, the estimated memory cost of HEOM is several orders of magnitude higher than that of DAMPF.

## Supplementary Materials

### This PDF file includes:

Supplementary Text Figs. S1 to S6 Tables S1 to S5 References
