# Supplementary material for: Full microscopic simulations uncover persistent quantum effects in primary photosynthesis
Source: Sci Adv. 2025 Oct 1;11(40):eady6751. doi: 10.1126/sciadv.ady6751 (PMC13155558; doi:10.1126/sciadv.ady6751)
Supplement: Supplementary file 1 — Supplementary Text Figs. S1 to S6 Tables S1 to S5 References [file sciadv.ady6751_sm.pdf]

Science Advances
AAAS

Supplementary Materials for

Full microscopic simulations uncover persistent quantum effects in primary photosynthesis

Nicola Lorenzoni *et al.*

Corresponding author: Susana F. Huelga, susana.huelga@uni-ulm.de; Martin B. Plenio, martin.plenio@uni-ulm.de

Sci. Adv. **11**, eady6751 (2025)
DOI: 10.1126/sciadv.ady6751

This PDF file includes:

Supplementary Text
Figs. S1 to S6
Tables S1 to S5
References

Supplementary Text

Phenomenological conditions for long-lived excitonic coherences

Here we discuss the conditions for long-lived excitonic coherences observed in numerically exact simulations of the FMO complex.

The interaction between exciton states and underdamped intra-pigment vibrational modes is decomposed into two parts $H_{e-v} = H_{e-v}^{(d)} + H_{e-v}^{(o)}$, where $H_{e-v}^{(d)}$ denotes the diagonal terms in the exciton basis

$$H_{e-v}^{(d)} = \sum_{i=1}^{7} |E_i\rangle\langle E_i| \sum_{n=1}^{7}\sum_{k=1}^{62} |\langle E_i|\epsilon_n\rangle|^2 \omega_k \sqrt{s_k}(b_{n,k} + b_{n,k}^{\dagger}), \quad \text{(S1)}$$

and $H_{e-v}^{(o)}$ represents the off-diagonal terms in the exciton basis

$$H_{e-v}^{(o)} = \sum_{i\neq j}^{7} |E_i\rangle\langle E_j| \sum_{n=1}^{7}\sum_{k=1}^{62} \langle E_i|\epsilon_n\rangle\langle\epsilon_n|E_j\rangle \omega_k \sqrt{s_k}(b_{n,k} + b_{n,k}^{\dagger}). \quad \text{(S2)}$$

The off-diagonal vibronic couplings $H_{e-v}^{(o)}$ induce transitions between different exciton states while creating or annihilating vibrational excitations. Roughly speaking, if an exciton state $|E_j, 0\rangle$ is initially populated with $|0\rangle$ denoting the global vibrational ground state of the 62 intra-pigment modes, the off-diagonal vibronic couplings $H_{e-v}^{(o)}$ induce a transition to $|\psi(t)\rangle = \alpha_0(t)|E_j, 0\rangle + \sum_{k=1}^{62} \alpha_k(t)|E_i, 1_k\rangle$ over time t, where $|1_k\rangle$ denotes a singly-excited vibrational state of the k-th intra-pigment mode while all other modes remain in their vibrational ground states. This results in vibronically-generated excitonic coherences given by $\mathrm{Tr}_v[\langle E_i|\psi(t)\rangle\langle\psi(t)|E_j\rangle]$ where Tr_v denotes the partial trace over vibrational degrees of freedom. Since the global vibrational ground state of $|E_j, 0\rangle$ and the singly-excited vibrational states of $|E_i, 1_k\rangle$ are defined with respect to different vibrational potential surfaces conditioned on the electronic states $|E_j\rangle$ or $|E_i\rangle$, described by the diagonal vibronic couplings $H_{e-v}^{(d)}$, the overlaps $\langle 0|1_k\rangle$ between these vibrational states do not vanish, leading to non-zero excitonic coherence amplitudes. The strength of the off-diagonal vibronic coupling responsible for exciton state transitions $|E_i\rangle \leftrightarrow |E_j\rangle$ depends on the spatial overlap $\beta_{(i,j)} = \sum_{n=1}^{7} |\langle E_i|\epsilon_n\rangle\langle\epsilon_n|E_j\rangle|$ between the exciton states.

In case of the FMO complex, which supports more than two exciton states, the previous explanation is insufficient to describe the vibronically-generated excitonic coherence dynamics. If an exciton state $|E_j, 0\rangle$ is initially populated and multiple transitions occur consecutively from $|E_j\rangle$ to $|E_i\rangle$, and then to $|E_f\rangle$, the total system at time t may be described by $|\psi(t)\rangle = \alpha_0(t)|E_j, 0\rangle + \sum_{k=1}^{62} \alpha_k(t)|E_i, 1_k\rangle + \sum_{k=1}^{62} \alpha'_k(t)\, |E_f, 2_k\rangle$. As the amplitudes $\alpha_k(t)$ responsible for excitonic coherences between $|E_i\rangle$ and $|E_j\rangle$ decrease due to further transitions from $|E_i\rangle$ to $|E_f\rangle$, this effectively induces the dephasing of excitonic coherence $\langle E_i|\rho(t)|E_j\rangle$ via a relaxation process. Therefore, long-lived oscillations of dynamically-generated excitonic coherences are expected when at least one of the exciton states, $|E_i\rangle$ or $|E_j\rangle$, is either the lowest-energy exciton state $|E_1\rangle$ or a meta-stable state.

For the electronic parameters of the FMO complex estimated in Ref. (*3*) and summarized in table S1, fig. S3A presents the population dynamics of each exciton state $|E_k\rangle$ when initially populated (i.e., $\rho(0) = |E_k\rangle\langle E_k|$). Notably, the populations of the lowest-energy exciton $|E_1\rangle$

and the exciton state $|E_3\rangle$, which is an electronic eigenstate localized on a quasi-dimeric unit of sites 1 and 2 with a lower exciton energy $E_3 < E_6$ (see fig. S3B), remain high for up to 1 ps, compared to other exciton states. This suggests that $|E_3\rangle$ is a meta-stable state. Figure S3C shows that relatively large spatial overlaps $\beta_{(i,j)}$ occur between multiple exciton pairs (i,j) (see purple-yellow scale), but only the (1,2) and (3,6) pairs involve either the lowest-energy exciton $|E_1\rangle$ or the meta-stable state $|E_3\rangle$. For these pairs, long-lived excitonic coherences exhibit relatively large amplitudes (see red-yellow scale).

In figs. S3D-F, we modified site energies so that no quasi-dimeric units are formed in the FMO complex using the parameters summarized in the first row of table S5. In this case, we found that there is no meta-stable state, and as a result, only the (1,2) and (1,3) pairs exhibit relatively large overlaps $\beta_{(i,j)}$ and involve the lowest-energy exciton state $|E_1\rangle$. These pairs exhibit relatively large amplitudes of long-lived excitonic coherences. Similarly, in figs. S3G-I, we considered a different set of site energies that introduce three quasi-dimeric units in the FMO complex (see the second row of table S5). Here, the exciton state $|E_6\rangle$ becomes more stable and has a larger spatial overlap $\beta_{(6,7)}$ with the highest-energy exciton state $|E_7\rangle$, leading to an enhanced amplitude of the long-lived excitonic coherence between $|E_6\rangle$ and $|E_7\rangle$.

Amplitude maps of long-lived oscillations from rephasing ground-state bleaching and stimulated emission signals

For a given pair of excitation and detection frequencies (ω_1, ω_3), we decomposed the rephasing 2D signals along the waiting time t_2 into damped oscillations and exponential decay terms. The non-oscillatory exponential components were removed using a high-pass filter with a frequency cutoff $100\ \mathrm{cm}^{-1}$, which is lower than the excitonic splitting $\Delta_{12} \approx 283\ \mathrm{cm}^{-1}$ of the dimeric PPC model used in 2D simulations, as well as the vibrational frequencies of the intra-pigment modes summarized in table S3. To identify the spectral regions (ω_1, ω_3) where the amplitudes of long-lived oscillations are maximized, we applied the high-pass filter to ground-state bleaching (GSB) and stimulated emission (SE) signals, separately. We then integrated the absolute value of the high-pass filtered signals over a time window $t_2 \in [0.2, 1.0]$ ps, as shown in figs. S5A and B. The three peak positions, P1, P2 and P3, in fig. S5B of the rephasing SE spectra were considered in Fig. 5B of the main text.

Frequency spectra of rephasing ground-state bleaching and stimulated emission signals

In this work, we non-perturbatively computed rephasing 2D spectra of a dimeric system with each site subject to the FMO phonon spectral density. For six representative spectral positions P1-P6 marked in fig. S6A (see black circles), we present the frequency spectra of the SE and GSB signals in figs. S6B-G. More specifically, for given excitation and detection frequencies (ω_1, ω_3), the real parts of the SE and GSB signals were Fourier transformed with respect to waiting time t_2, yielding the frequency spectra as functions of beating frequency ω_2. To examine the magnitude of 2D oscillations, the absolute values of the resulting spectra were analyzed. Notably, the GSB signals exhibit multiple narrow peaks below $1600\ \mathrm{cm}^{-1}$, with peak positions that closely match those of the Huang–Rhys factor spectrum of the FMO complex, defined as $s(\omega) = J(\omega)/\omega^2$, where $J(\omega)$ denotes the phonon spectral density. This indicates that the oscillations in the GSB signals are dominated by single-phonon vibrational coherences, as the small Huang-Rhys factors of the intra-pigment modes of bacteriochlorophylls (see table S3) result in negligible two-phonon transitions. By contrast, the SE signals show multiple peaks beyond the vibrational frequency range of the intra-pigment modes (i.e., $\omega_2 \gg$

$1600\ \mathrm{cm}^{-1}$). This suggests that the oscillatory signals in this high-frequency regime are dominated by excited-state coherences in the SE signals. To confirm that this feature is not sensitive to the choice of excitation and detection frequencies (ω_1, ω_3), integrated frequency spectra over two areas indicated in fig. S6A (see red squares) are shown in figs. S6H and I.

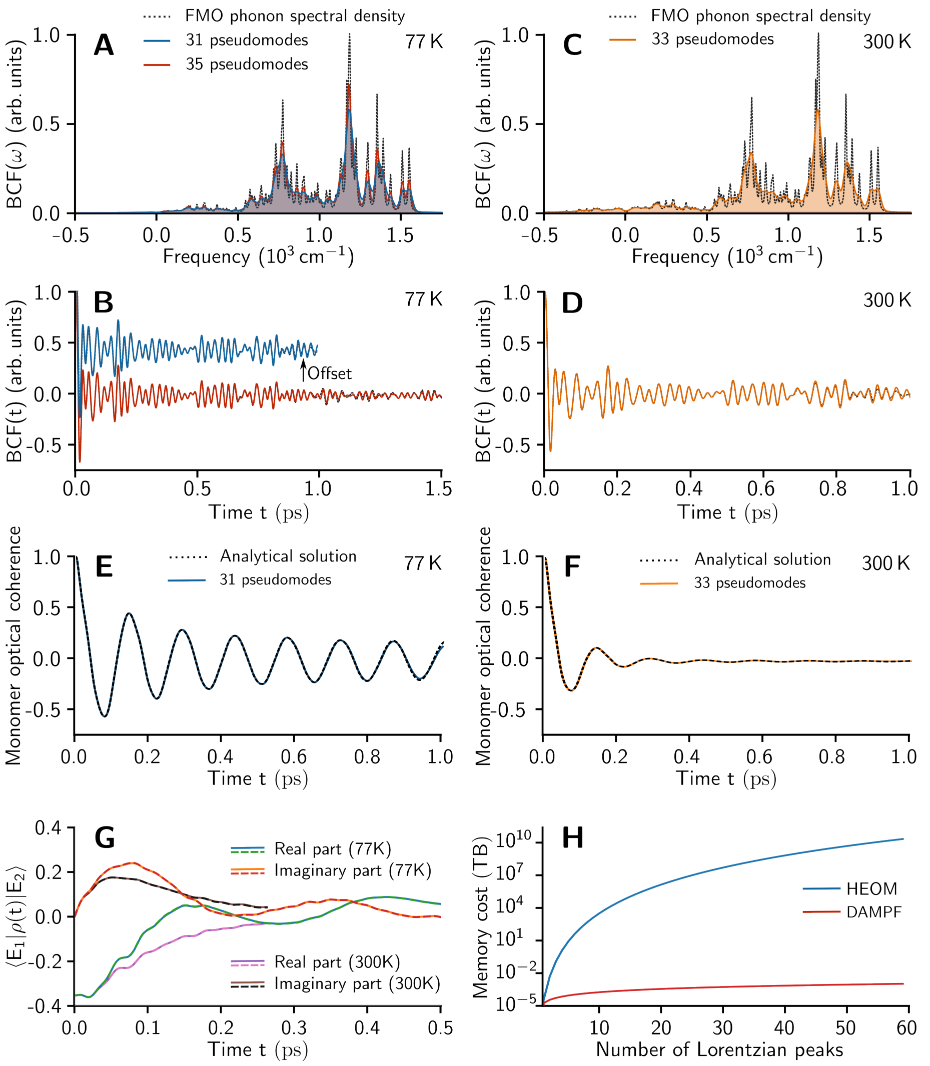

Fig. S1. Systematic coarse-graining of the environment.

(**A**) The frequency spectra of the bath correlation functions (BCFs) of the effective environments modeled using 31 and 35 pseudomodes are shown in blue and red, respectively, compared with the FMO phonon spectral density (SD) shown as a black dashed line. (**B**) The effective environments quantitatively reproduce the BCF of the FMO SD at 77 K up to 1.0 ps and 1.5 ps, respectively. (**C**) The frequency spectrum of the BCF of the effective environment modeled using 33 pseudomodes, including two negative-frequency modes, is shown in orange. (**D**) This effective environment can quantitatively reproduce the BCF of the FMO SD at 300 K up to 1.0 ps. Numerically exact optical coherence dynamics of a monomer computed by DAMPF, based on the pseudomode parameters, are well matched with the analytical solutions of the monomer dynamics at (**E**) 77 K and (**F**) 300 K. (**G**) Comparison of excitonic coherence dynamics of a dimeric system computed using DAMPF and T-TEDOPA. (**H**) Comparison of memory costs for HEOM and DAMPF as a function of the number of Lorentzian peaks in the phonon spectral density for a seven-site PPC model.

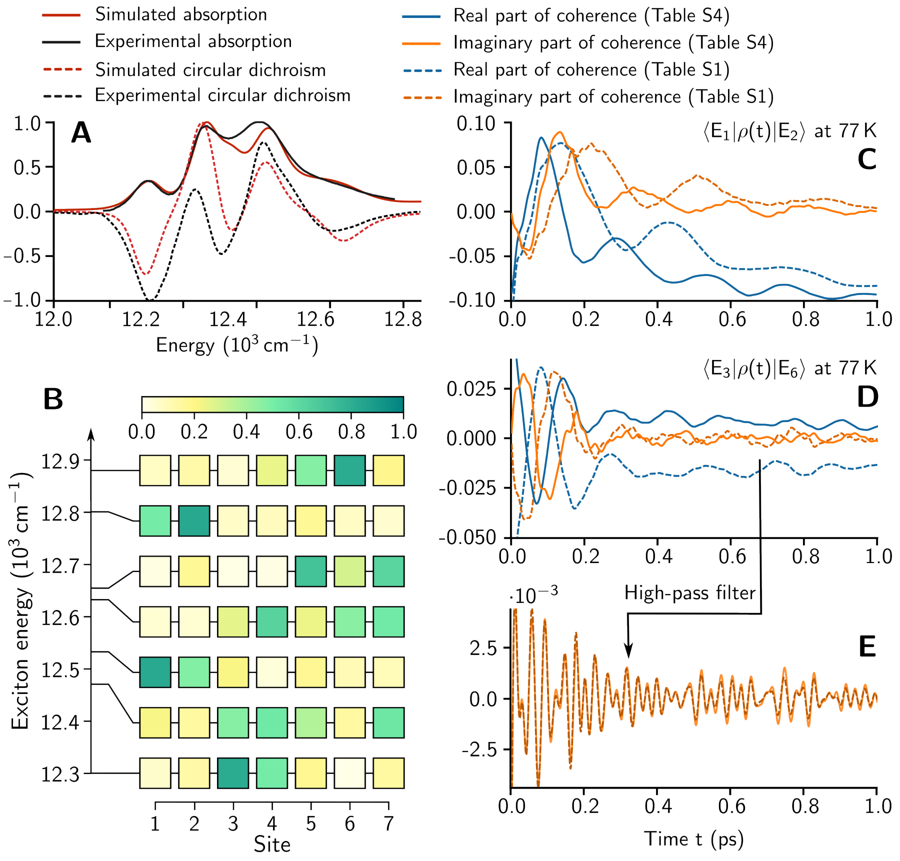

Fig. S2. Refined electronic parameters of the FMO complex and excitonic coherence dynamics.

(**A**) Numerically exact absorption and circular dichroism spectra of the FMO complex at 77 K, computed using the electronic parameters in table S4 and the experimentally estimated phonon spectral density of the FMO complex, are shown in red. These computed spectra closely match the experimental data (*53*) shown in black. (**B**) Population distributions of the seven exciton states $|E_k\rangle$ of the FMO complex in the site basis, computed using the refined electronic parameters in table S4. (**C**) The excitonic coherence dynamics at 77 K between $|E_1\rangle$ and $|E_2\rangle$ and (**D**) those between $|E_3\rangle$ and $|E_6\rangle$, simulated using the refined electronic parameters, are shown as solid lines. For comparison with Fig. 3 of the main text, the excitonic coherence dynamics computed using the electronic parameters estimated in Ref. (*3*) are shown in dashed lines. (**E**) The high-frequency long-lived components of the excitonic coherences between $|E_3\rangle$ and $|E_6\rangle$, extracted via a high-pass filter, are nearly identical for both sets of electronic parameters.

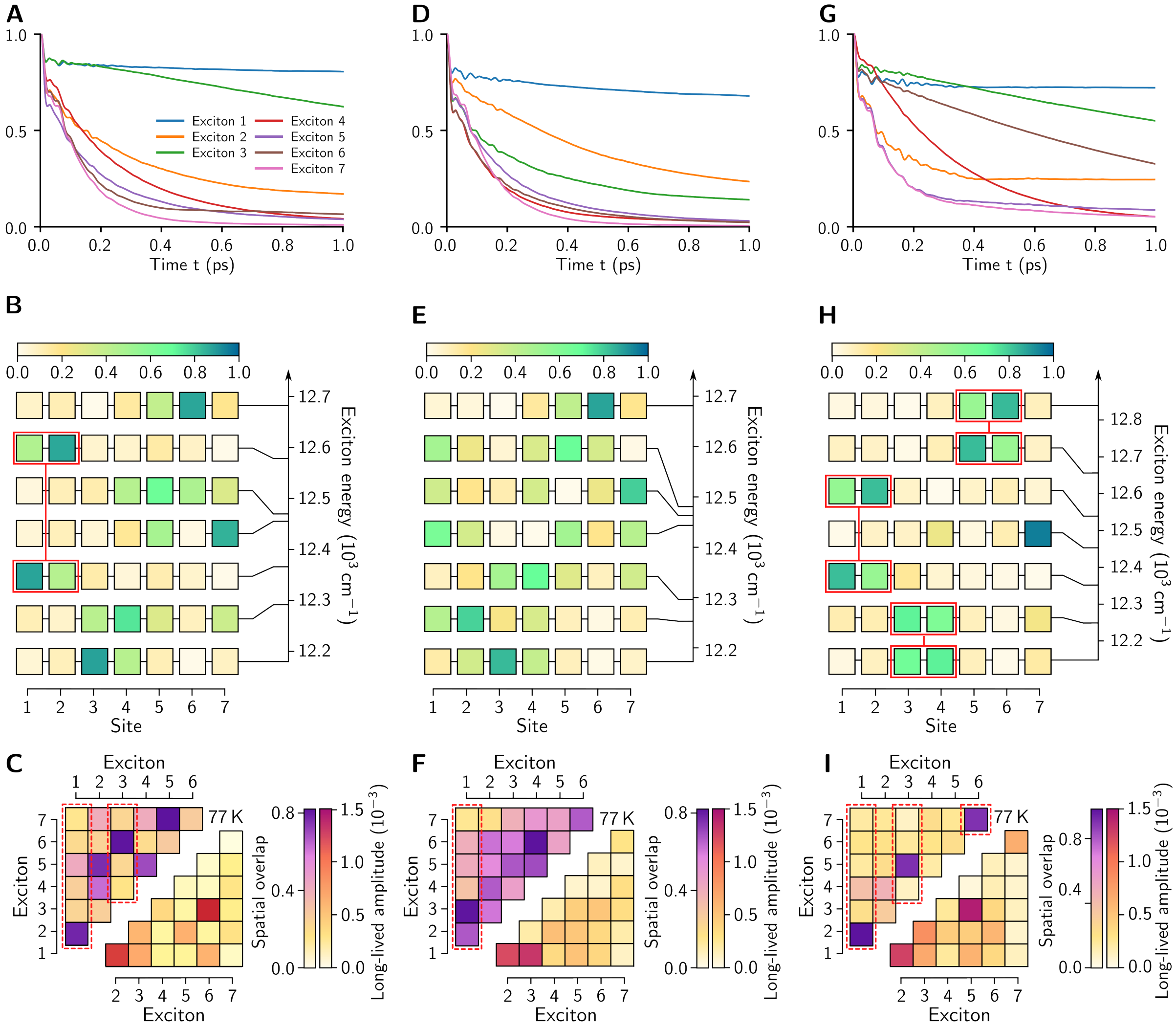

Fig. S3. Exciton population dynamics and phenomenological conditions for long-lived excitonic coherences.

(**A**) Population dynamics of exciton state $|E_k\rangle$ when initially populated (i.e., $\rho(0) = |E_k\rangle\langle E_k|$) computed using the electronic parameters estimated in Ref. (*3*) (see table S1). (**B**) Population distributions of the seven exciton states $|E_k\rangle$ in the site basis. (**C**) Spatial overlaps $\beta_{(i,j)}$ between exciton states (purple-yellow scale) and the amplitudes of long-lived excitonic coherences (red-yellow scale). (**D-F**) Results obtained using the first set of site energies from table S5, where no quasi-dimeric units are formed. (**G-I**) Results obtained using the second set of site energies from table S5, where three quasi-dimeric units are formed. In all simulations, the FMO phonon spectral density at $T = 77$ K was considered.

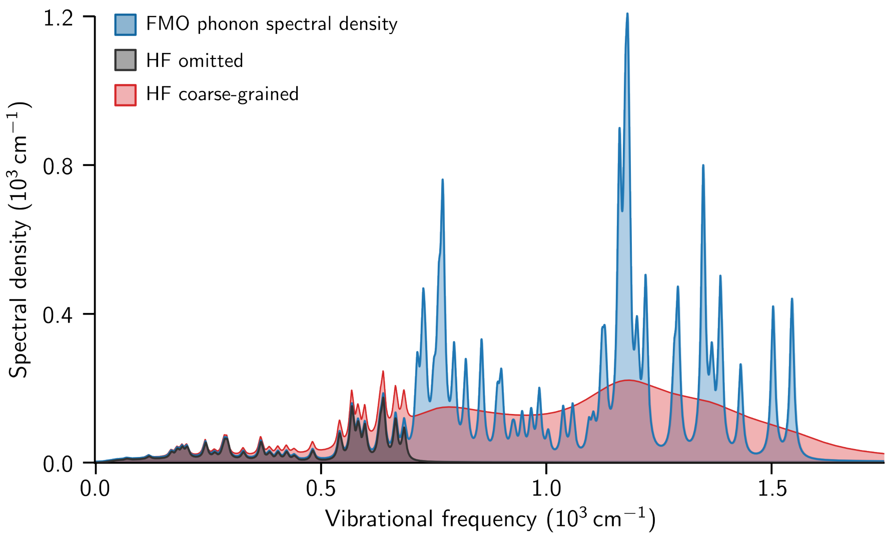

Fig. S4. Approximated high-frequency intra-pigment modes.

The model spectral densities, in which high-frequency (HF) intra-pigment modes with vibrational frequencies $\omega_k \geq 700$ cm^{-1} are either omitted (black) or coarse-grained (red), are shown. The experimentally estimated FMO phonon spectral density is shown in blue.

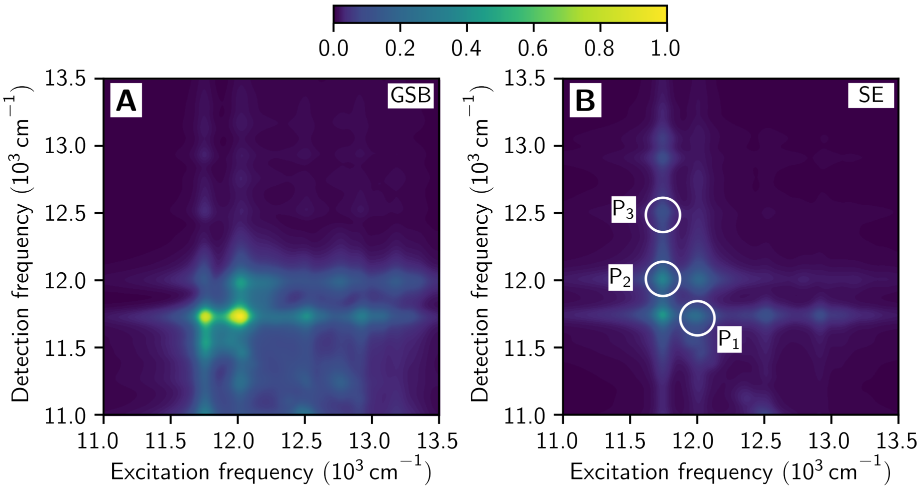

Fig. S5. Amplitude maps of long-lived oscillatory rephasing signals.

The absolute value of the real part of the oscillatory (**A**) GSB and (**B**) SE signals, obtained via a high-pass filter, was integrated over the waiting time $t_2 \in [0.2, 1.0]$ ps for each spectral position (ω_1, ω_3). A dimeric PPC model ($\epsilon_1 - \epsilon_2 = 200$ cm^{-1}, $V_{12} = 100$ cm^{-1}, orthogonal transition dipole moments, $\boldsymbol{\mu_1} \cdot \boldsymbol{\mu_2} = 0$, with identical magnitudes, $\boldsymbol{\mu_1} \cdot \boldsymbol{\mu_1} = \boldsymbol{\mu_2} \cdot \boldsymbol{\mu_2}$) was considered with the FMO phonon spectral density at 77 K.

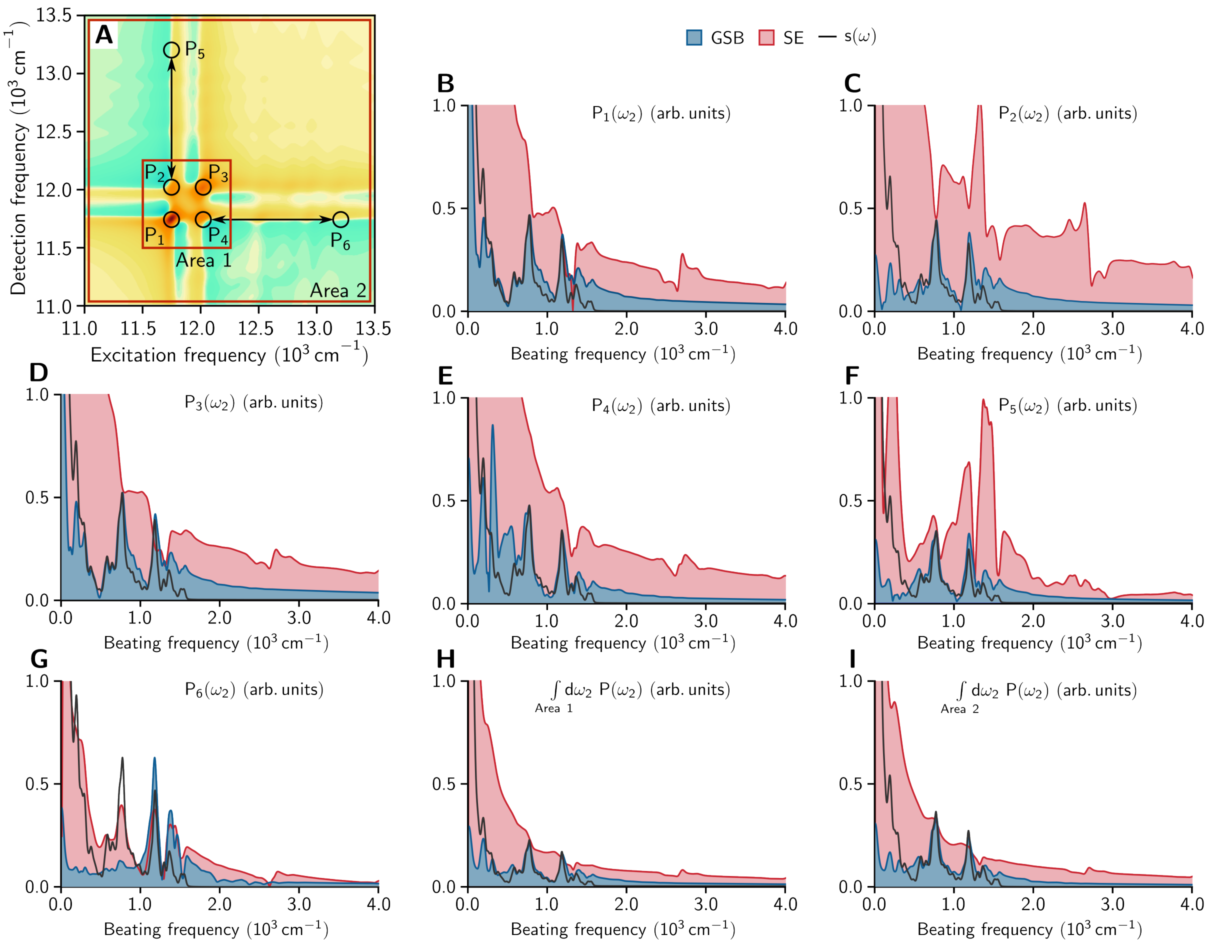

Fig. S6. Frequency spectra of SE and GSB signals.
(**A**) Rephasing spectra at zero waiting time, $t_2 = 0$, in which six spectral positions P1-P6 (black circles) and spectral areas 1 and 2 (red squares) are highlighted. (**B-G**) Absolute values of the Fourier-transformed spectra of the real parts of the SE and GSB signals at the six spectral positions, shown in red and blue, respectively. The Huang-Rhys factor spectrum $s(\omega)$ of the FMO complex, shown in black. (**H-I**) Integrated frequency spectra over the areas 1 and 2.

Table S1. Electronic Hamiltonian of the FMO complex, estimated in Ref. (*3*).

$\langle n\|H_e\|m\rangle$ [cm^{-1}]	1	2	3	4	5	6	7
1	12410	-87.7	5.5	-5.9	6.7	-13.7	-9.9
2		12530	30.8	8.2	0.7	11.8	4.3
3			12210	-53.5	-2.2	-9.6	6.0
4				12320	-70.7	-17.0	-63.3
5					12480	81.1	-1.3
6						12630	39.7
7							12440

Table S2. Transition dipole moments of the seven pigments in the FMO complex.

The parameters are given in arbitrary units, as only relative values are relevant in this work.

$\boldsymbol{\mu}_n$	1	2	3	4	5	6	7
x-component	-0.741	-0.857	-0.197	-0.799	-0.740	-0.135	-0.495
y-component	-0.561	0.504	0.957	-0.534	0.656	-0.879	-0.708
z-component	-0.370	-0.107	-0.211	-0.277	0.164	0.457	-0.503

Table S3. The vibrational frequencies ω_k and the Huang-Rhys factors s_k of the 62 intra-pigment modes of the FMO complex, estimated in Ref. (*9*).

k	1	2	3	4	5	6	7	8	9	10
ω_k [cm^{-1}]	46	68	117	167	180	191	202	243	263	284
s_k	0.011	0.011	0.009	0.009	0.010	0.011	0.011	0.012	0.003	0.008
k	11	12	13	14	15	16	17	18	19	20
ω_k [cm^{-1}]	291	327	366	385	404	423	440	481	541	568
s_k	0.008	0.003	0.006	0.002	0.002	0.002	0.001	0.002	0.004	0.007
k	21	22	23	24	25	26	27	28	29	30
ω_k [cm^{-1}]	582	597	630	638	665	684	713	726	731	750
s_k	0.004	0.004	0.003	0.006	0.004	0.003	0.007	0.010	0.005	0.004
k	31	32	33	34	35	36	37	38	39	40
ω_k [cm^{-1}]	761	770	795	821	856	891	900	924	929	946
s_k	0.009	0.018	0.007	0.006	0.007	0.003	0.004	0.001	0.001	0.002
k	41	42	43	44	45	46	47	48	49	50
ω_k [cm^{-1}]	966	984	1004	1037	1058	1094	1104	1123	1130	1162
s_k	0.002	0.003	0.001	0.002	0.002	0.001	0.001	0.003	0.003	0.009
k	51	52	53	54	55	56	57	58	59	60
ω_k [cm^{-1}]	1175	1181	1201	1220	1283	1292	1348	1367	1386	1431
s_k	0.007	0.010	0.003	0.005	0.002	0.004	0.007	0.002	0.004	0.002
k	61	62								
ω_k [cm^{-1}]	1503	1545								
s_k	0.003	0.003								

Table S4. Refined electronic Hamiltonian of the FMO complex, obtained by fitting numerically exact absorption and circular dichroism spectra to experimental data at 77 K (see fig. S2A).

$\langle n \vert H_e \vert m \rangle$ [cm^{-1}]	1	2	3	4	5	6	7
1	12598	-120.0	8.2	-8.5	10.0	-13.1	-16.1
2		12719	46.8	16.3	1.7	12.9	9.7
3			12372	-99.8	-13.0	-2.8	6.1
4				12515	-93.0	-38.1	-80.0
5					12664	102.5	-31.9
6						12794	53.8
7							12593

Table S5. Modified site energies of the FMO complex, corresponding to cases where no quasi-dimeric units or three quasi-dimeric units are formed (see figs. S3D-F and G-I, respectively).

ϵ_n [cm^{-1}]	1	2	3	4	5	6	7
No quasi-dimeric units	12410	12290	12210	12320	12480	12630	12440
Three quasi-dimeric units	12410	12480	12210	12220	12700	12780	12440

REFERENCES AND NOTES

1. R. E. Blankenship, *Molecular Mechanisms of Photosynthesis* (Wiley-Blackwell, 2002).

2. S. I. E. Vulto, M. A. de Baat, R. J. W. Louwe, H. P. Permentier, T. Neef, M. Miller, H. van Amerongen, T. J. Aartsma, Exciton simulations of optical spectra of the FMO complex from the green sulfur bacterium Chlorobium tepidum at 6 K. *J. Phys. Chem. B* **102**, 9577–9582 (1998).

3. J. Adolphs, T. Renger, How proteins trigger excitation energy transfer in the FMO complex of green sulfur bacteria. *Biophys. J.* **91**, 2778–2797 (2006).

4. M. S. A. Busch, F. Müh, M. E.-A. Madjet, T. Renger, The eighth bacteriochlorophyll completes the excitation energy funnel in the FMO protein. *J. Phys. Chem. Lett.* **2**, 93–98 (2011).

5. T. Renger, A. Klinger, F. Steinecker, M. S. A. Busch, J. Numata, F. Müh, Normal mode analysis of the spectral density of the Fenna-Matthews-Olson light-harvesting protein: How the protein dissipates the excess energy of excitons. *J. Phys. Chem. B* **116**, 14565–14580 (2012).

6. M. K. Lee, D. F. Coker, Modeling electronic-nuclear interactions for excitation energy transfer processes in light-harvesting complexes. *J. Phys. Chem. Lett.* **7**, 3171–3178 (2016).

7. C. W. Kim, B. Choi, Y. M. Rhee, Excited state energy fluctuations in the Fenna-Matthews-Olson complex from molecular dynamics simulations with interpolated chromophore potentials. *Phys. Chem. Chem. Phys.* **20**, 3310–3319 (2018).

8. S. Maity, U. Kleinekathöfer, Recent progress in atomistic modeling of light-harvesting complexes: A mini review. *Photosynth. Res.* **156**, 147–162 (2023).

9. M. Rätsep, A. Freiberg, Electron phonon and vibronic couplings in the FMO bacteriochlorophyll a antenna complex studied by difference fluorescence line narrowing. *J. Lumin.* **127**, 251–259 (2007).

10. F. Caycedo-Soler, A. Mattioni, J. Lim, T. Renger, S. F. Huelga, M. B. Plenio, Exact simulation of pigment-protein complexes unveils vibronic renormalization of electronic parameters in ultrafast spectroscopy. *Nat. Commun.* **13**, 2912 (2022).

11. A. Strathearn, P. Kirton, D. Kilda, J. Keeling, B. W. Lovett, Efficient non-Markovian quantum dynamics using time-evolving matrix product operators. *Nat. Commun.* **9**, 3322 (2018).

12. D. Tamascelli, A. Smirne, J. Lim, S. F. Huelga, M. B. Plenio, Efficient simulation of finite-temperature open quantum systems. *Phys. Rev. Lett.* **123**, 090402 (2019).

13. A. D. Somoza, O. Marty, J. Lim, S. F. Huelga, M. B. Plenio, Dissipation-assisted matrix product factorization. *Phys. Rev. Lett.* **123**, 100502 (2019).

14. M. Cygorek, M. Cosacchi, A. Vagov, V. M. Axt, B. W. Lovett, J. Keeling, E. M. Gauger, Simulation of open quantum systems by automated compression of arbitrary environments. *Nat. Phys.* **18**, 662–668 (2022).

15. N. Lorenzoni, N. Cho, J. Lim, D. Tamascelli, S. F. Huelga, M. B. Plenio, Systematic coarse graining of environments for the nonperturbative simulation of open quantum systems. *Phys. Rev. Lett.* **132**, 100403 (2024).

16. A. Ishizaki, G. R. Fleming, Theoretical examination of quantum coherence in a photosynthetic system at physiological temperature. *Proc. Natl. Acad. Sci. U.S.A.* **106**, 17255–17260 (2009).

17. P. Nalbach, D. Braun, M. Thorwart, Exciton transfer dynamics and quantumness of energy transfer in the Fenna-Matthews-Olson complex. *Phys. Rev. E* **84**, 041926 (2011).

18. C. Kreisbeck, T. Kramer, Long-lived electronic coherence in dissipative exciton dynamics of light-harvesting complexes. *J. Phys. Chem. Lett.* **3**, 2828–2833 (2012).

19. S. M. Blau, D. I. G. Bennett, C. Kreisbeck, G. D. Scholes, A. Aspuru-Guzik, Local protein solvation drives direct down-conversion in phycobiliprotein PC645 via incoherent vibronic transport. *Proc. Natl. Acad. Sci. U.S.A.* **115**, E3342–E3350 (2018).

20. Y. Tanimura, R. Kubo, Time evolution of a quantum system in contact with a nearly Gaussian-Markoffian noise bath. *J. Physical Soc. Japan* **58**, 101–114 (1989).

21. D. M. Jonas, Two-dimensional femtosecond spectroscopy. *Annu. Rev. Phys. Chem.* **54**, 425–463 (2003).

22. T. Brixner, T. Mančal, I. V. Stiopkin, G. R. Fleming, Phase-stabilized two-dimensional electronic spectroscopy. *J. Chem. Phys.* **121**, 4221–4236 (2004).

23. V. Butkus, D. Zigmantas, L. Valkunas, D. Abramavicius, Vibrational vs. electronic coherences in 2D spectrum of molecular systems. *Chem. Phys. Lett.* **545**, 40–43 (2012).

24. V. Tiwari, W. K. Peters, D. M. Jonas, Electronic resonance with anticorrelated pigment vibrations drives photosynthetic energy transfer outside the adiabatic framework. *Proc. Natl. Acad. Sci. U.S.A.* **110**, 1203–1208 (2013).

25. M. B. Plenio, J. Almeida, S. F. Huelga, Origin of long-lived oscillations in 2D-spectra of a quantum vibronic model: Electronic versus vibrational coherence. *J. Chem. Phys.* **139**, 235102 (2013).

26. H.-G. Duan, V. I. Prokhorenko, R. J. Cogdell, K. Ashraf, A. L. Stevens, M. Thorwart, R. J. D. Miller, Nature does not rely on long-lived electronic quantum coherence for photosynthetic energy transfer. *Proc. Natl. Acad. Sci. U.S.A.* **114**, 8493–8498 (2017).

27. E. Thyrhaug, R. Tempelaar, M. J. P. Alcocer, K. Žídek, D. Bína, J. Knoester, T. L. C. Jansen, D. Zigmantas, Identification and characterization of diverse coherences in the Fenna-Matthews-Olson complex. *Nat. Chem.* **10**, 780–786 (2018).

28. G. S. Engel, T. R. Calhoun, E. L. Read, T. Ahn, T. Mančal, Y. Cheng, R. E. Blankenship, G. R. Fleming, Evidence for wavelike energy transfer through quantum coherence in photosynthetic systems. *Nature* **446**, 782–786 (2007).

29. G. Panitchayangkoon, D. Hayes, K. A. Fransted, J. R. Caram, E. Harel, J. Wen, R. E. Blankenship, G. S. Engel, Long-lived quantum coherence in photosynthetic complexes at physiological temperature. *Proc. Natl. Acad. Sci. U.S.A.* **107**, 12766–12770 (2010).

30. J. Cao, R. J. Cogdell, D. F. Coker, H.-G. Duan, J. Hauer, U. Kleinekathöfer, T. L. C. Jansen, T. Mančal, R. J. D. Miller, J. P. Ogilvie, V. I. Prokhorenko, T. Renger, H.-S. Tan, R. Tempelaar, M. Thorwart, E. Thyrhaug, S. Westenhoff, D. Zigmantas, Quantum biology revisited. *Sci. Adv.* **6**, eaaz4888 (2020).

31. M. Maiuri, E. E. Ostroumov, R. G. Saer, R. E. Blankenship, G. D. Scholes, Coherent wavepackets in the Fenna-Matthews-Olson complex are robust to excitonic-structure perturbations caused by mutagenesis. *Nat. Chem.* **10**, 177–183 (2018).

32. H. Breuer, F. Petruccione, *The Theory of Open Quantum Systems* (Oxford Univ. Press, 2003).

33. A. Kolli, E. J. O'Reilly, G. D. Scholes, A. Olaya-Castro, The fundamental role of quantized vibrations in coherent light harvesting by cryptophyte algae. *J. Chem. Phys.* **137**, 174109 (2012).

34. A. W. Chin, J. Prior, R. Rosenbach, F. Caycedo-Soler, S. F. Huelga, M. B. Plenio, The role of non-equilibrium vibrational structures in electronic coherence and recoherence in pigment-protein complexes. *Nat. Phys.* **9**, 113–118 (2013).

35. M. Cho, *Two-Dimensional Optical Spectroscopy* (CRC Press, 2009).

36. J. Lim, C. M. Bösen, A. D. Somoza, C. P. Koch, M. B. Plenio, S. F. Huelga, Multi-color quantum control for suppressing ground state coherences in two-dimensional electronic spectroscopy. *Phys. Rev. Lett.* **123**, 233201 (2019).

37. V. Zazubovich, I. Tibe, G. J. Small, Bacteriochlorophyll a Franck-Condon factors for the $S_0 \rightarrow S_1(Q_y)$ transition. *J. Phys. Chem. B* **105**, 12410–12417 (2001).

38. V. R. Policht, A. Niedringhaus, J. P. Ogilvie, Characterization of vibrational coherence in monomeric bacteriochlorophyll a by two-dimensional electronic spectroscopy. *J. Phys. Chem. Lett.* **9**, 6631–6637 (2018).

39. S. Irgen-Gioro, A. P. Spencer, W. O. Hutson, E. Harel, Coherences of bacteriochlorophyll a uncovered using 3D-electronic spectroscopy. *J. Phys. Chem. Lett.* **9**, 6077–6081 (2018).

40. E. Meneghin, D. Pedron, E. Collini, Characterization of the coherent dynamics of bacteriochlorophyll a in solution. *Chem. Phys.* **519**, 85–91 (2019).

41. S. S. Senlik, V. R. Policht, J. P. Ogilvie, Two-color nonlinear spectroscopy for the rapid acquisition of coherent dynamics. *J. Phys. Chem. Lett.* **6**, 2413–2420 (2015).

42. T. A. A. Olivera, N. H. C. Lewise, G. R. Fleming, Correlating the motion of electrons and nuclei with two-dimensional electronic-vibrational spectroscopy. *Proc. Natl. Acad. Sci. U.S.A.* **111**, 10061–10066 (2014).

43. T. L. Courtney, Z. W. Fox, K. M. Slenkamp, M. Khalil, Two-dimensional vibrational-electronic spectroscopy. *J. Chem. Phys.* **43**, 154201 (2015).

44. D. Kern-Michler, C. Neumann, N. Mielke, L. J. G. W. van Wilderen, M. Reinfelds, J. von Cosel, F. Santoro, A. Heckel, I. Burghardt, J. Bredenbeck, Controlling photochemistry via isotopomers and IR pre-excitation. *J. Am. Chem. Soc.* **140**, 926–931 (2018).

45. A. D. Somoza, N. Lorenzoni, J. Lim, S. F. Huelga, M. B. Plenio, Driving force and nonequilibrium vibronic dynamics in charge separation of strongly bound electron-hole pairs. *Commun. Phys.* **6**, 65 (2023).

46. D. Tamascelli, A. Smirne, S. F. Huelga, M. B. Plenio, Nonperturbative treatment of non-Markovian dynamics of open quantum systems. *Phys. Rev. Lett.* **120**, 030402 (2018).

47. J. Prior, A. W. Chin, S. F. Huelga, M. B. Plenio, Efficient simulation of strong system-environment interactions. *Phys. Rev. Lett.* **105**, 050404 (2010).

48. T. Lacroix, B. Le Dé, A. Riva, A. J. Dunnett, A. W. Chin, MPSDynamics.jl: Tensor network simulations for finite-temperature (non-Markovian) open quantum system dynamics. *J. Chem. Phys.* **161**, 084116 (2024).

49. H. M. Berman, J. Westbrook, Z. Feng, G. Gilliland, T. N. Bhat, H. Weissig, I. N. Shindyalov, P. E. Bourne, The Protein Data Bank. *Nucleic Acids Res.* **28**, 235–242 (2000).

50. D. E. Tronrud, J. Wen, L. Gay, R. E. Blankenship, The structural basis for the difference in absorbance spectra for the FMO antenna protein from various green sulfur bacteria. *Photosynth. Res.* **100**, 79–87 (2009).

51. D. E. Tronrud, A. Camara-Artigas, R. E. Blankenship, J. P. Allen, Crystal structure of the Fenna-Matthews-Olson protein from *Chlorobaculum tepidum* (2009); https://doi.org/10.2210/pdb3ENI/pdb.

52. D. Sehnal, S. Bittrich, M. Deshpande, R. Svobodová, K. Berka, V. Bazgier, S. Velankar, S. K. Burley, J. Koča, A. S. Rose, Mol* viewer: Modern web app for 3D visualization and analysis of large biomolecular structures. *Nucleic Acids Res.* **49**, W431–W437 (2021).

53. A. N. Melkozernov, J. M. Olson, Y.-F. Li, J. P. Allen, R. E. Blankenship, Orientation and excitonic interactions of the Fenna-Matthews-Olson bacteriochlorophyll a protein in membranes of the green sulfur bacterium Chlorobium tepidum. *Photosynth. Res.* **56**, 315–328 (1998).
